# Inflammatory profile in *LRRK2*-associated prodromal and clinical PD

**DOI:** 10.1186/s12974-016-0588-5

**Published:** 2016-05-24

**Authors:** Kathrin Brockmann, Anja Apel, Claudia Schulte, Nicole Schneiderhan-Marra, Claustre Pont-Sunyer, Dolores Vilas, Javier Ruiz-Martinez, Markus Langkamp, Jean-Christophe Corvol, Florence Cormier, Thomas Knorpp, Thomas O. Joos, Thomas Gasser, Birgitt Schüle, Jan O. Aasly, Tatiana Foroud, Jose Felix Marti-Masso, Alexis Brice, Eduardo Tolosa, Connie Marras, Daniela Berg, Walter Maetzler

**Affiliations:** Department of Neurodegenerative Diseases and Hertie Institute for Clinical Brain Research, University of Tübingen, Tübingen, Germany; German Center for Neurodegenerative Diseases (DZNE), Tübingen, Germany; Natural and Medical Sciences Institute at the University of Tübingen (NMI), Reutlingen, Germany; Parkinson’s Disease and Movement Disorders Unit, Neurology Service, Hospital Clinic de Barcelona, Universitat de Barcelona, Institutd’Investigacions Biomediques August Pi I Sunyer (IDIBAPS), Centro de Investigación Biomédica en Red sobre Enfermedades Neurodegenerativas (CIBERNED), Barcelona, Spain; Hospital Universitario Donostia, Biodonostia Institut, San Sebastián, Guipuzcoa Spain; Mediagnost GmbH, Reutlingen, Germany; Département de Génétique et Cytogénétique, INSERM, Sorbonne Universités, Hôpital de la Pitié Salpêtrière, Paris, France; Parkinson Institute and Clinical Center, 675 Almanor Ave, Sunnyvale, CA USA; Department of Neurology, St. Olavs Hospital, Trondheim, Norway; Department of Medical and Molecular Genetics, Indiana University, Bloomington, IN USA; Morton and Gloria Shulman Movement Disorders Centre and the Edmond J Safra Program in Parkinson’s disease, Toronto Western Hospital, University of Toronto, Toronto, Canada

**Keywords:** Parkinson, *LRRK2*, Immune, Inflammation

## Abstract

**Background:**

There is evidence for a relevant role of inflammation in the pathogenesis of Parkinson’s disease (PD). Mutations in the *LRRK2* gene represent the most frequent genetic cause for autosomal dominant PD. LRRK2 is highly expressed in macrophages and microglia suggesting an involvement in inflammatory pathways. The objectives are to test (1) whether idiopathic PD and *LRRK2*-associated PD share common inflammatory pathways or present distinct profiles and (2) whether non-manifesting *LRRK2* mutation carriers present with similar aspects of inflammatory profiles as seen in PD-affected patients.

**Methods:**

We assessed serum profiles of 23 immune-associated markers and the brain-derived neurotrophic factor in 534 individuals from the MJFF *LRRK2* consortium.

**Results:**

A large proportion of inflammatory markers were gender-dependent. Both PD-affected cohorts showed increased levels of the pro-inflammatory marker fatty-acid-binding protein. Additionally, idiopathic PD but not *LRRK2*-associated PD patients showed increased levels of the pro-inflammatory marker interleukin-12-p40 as well as the anti-inflammatory species interleukin-10, brain-derived neurotrophic factor, and stem cell factor. Non-manifesting *LRRK2* mutation carriers including those with prodromal characteristics of PD presented with control-like inflammatory profiles.

**Conclusions:**

Concomitant inflammation seems to be associated with idiopathic and *LRRK2*-associated PD. Identifying PD patients in whom inflammatory processes play a major role in their pathophysiology might offer a new therapeutic window at least for a subgroup of patients. Since non-manifesting *LRRK2* mutation carriers with symptoms of the prodromal phase of PD did not show inflammatory profiles, activation of the immune system seems not an early event in the disease cascade.

**Electronic supplementary material:**

The online version of this article (doi:10.1186/s12974-016-0588-5) contains supplementary material, which is available to authorized users.

## Background

There is increasing evidence coming from post-mortem and biomarker analyses as well as genetic studies that inflammation is relevantly involved in the pathogenesis of Parkinson’s disease (PD) (reviewed in [1–8]). Although levels of cytokines and chemokines are highly variable, a substantial proportion of PD patients can show elevated levels of these proteins in serum and CSF (reviewed in [2]). This increase indicates an activation of the *innate* immune system with involvement of astrocytes and activation of microglia (reviewed in [1, 4]). Subtypes of astrocytes have recently been shown to effectively endocytose α-synuclein (asyn) species secreted from neurons and to produce glial inclusions and inflammatory responses [9].

Mutations in the gene for leucine-rich repeat kinase (*LRRK2*) represent the most frequent genetic cause associated with autosomal dominant PD. In general, the clinical phenotype of PD patients with *LRRK2* mutation (PD_LRRK2_) resembles late-onset idiopathic PD (IPD) with a good response to levodopa treatment. However, with respect to non-motor symptoms such as dementia and olfactory dysfunction, the clinical course in patients carrying the most common *LRRK2* mutation (p.G2019S) appears to be more benign than in IPD [10, 11]. There is increasing evidence for the involvement of LRRK2 in inflammatory pathways, linking PD_LRRK2_ to the immune system [3, 4]. LRRK2 is highly expressed in monocytes, macrophages, and microglia, and exposure to bacterial lipopolysaccharides (LPS) results in an up-regulation of LRRK2 protein as well as impaired autophagy in macrophages [4, 12]. Following this line, one could speculate that PD_LRRK2_ might be associated with distinct inflammatory cascades.

At present, it is not clear (1) whether idiopathic and *LRRK2*-associated PD share common inflammatory pathways or present with distinct profiles and (2) whether non-manifesting *LRRK2* mutation carriers (NMC_LRRK2_) present with similar aspects of inflammatory profiles as seen in PD-affected patients indicating an involvement of the immune system in earliest neurodegenerative processes. To evaluate these questions in this exploratory study, we applied an extensive battery of 27 immune-associated serum markers as well as the neurotrophin BDNF (which is directly secreted by activated monocytes in inflammatory brain lesions and promotes neuronal survival in vitro [13]) in a large multicenter cohort of the MJFF *LRRK2* consortium consisting of 534 individuals.

## Methods

### Centers and participants

In 2008, the Michael J. Fox Foundation established an international consortium to investigate the role of *LRRK2* in Parkinson’s disease (www.michaeljfox.org/page.html?lrrk2-cohort-consortium). The consortium brought together leading groups focusing on genetic forms of PD from nine countries across four continents (Canada, China, France, Germany, Israel, Norway, Spain, Tunisia, and the USA).

In total, clinical data and serum samples of 534 individuals from Canada, France, Germany, Norway, Spain, and the USA grouped into 4 cohorts were included in these cross-sectional analyses: (1) 144 IPD patients controlled to have no pathogenic mutation in the *LRRK2* gene, (2) 142 PD_LRRK2_, (3) 115 NMC_LRRK2_, and (4) 133 healthy control individuals controlled to have no pathogenic *LRRK2* mutation (CON); please see Additional file 1: Table S1 for the type of mutations and their distribution among the *LRRK2* cohorts.

Since PD_LRRK2_ is characterized by reduced penetrance and not all NMC_LRRK2_ will develop PD, we defined—in accordance with the new MDS research criteria for prodromal PD [14]—a “prodromal subgroup” of NMC_LRRK2_ individuals who may have a particularly high risk of conversion by carrying a genetic risk factor (*LRRK2* mutation) and additionally fulfilling at least two of the following prodromal criteria: mild motor impairment (*MDS-UPDRS-III* > 6), REM sleep behavior disorder (*RBD Questionnaire* ≥ 5), autonomic dysfunction (*SCOPA-AUT* ≥ 9), or hyposmia (age-related UPSIT cut-off [15]). Thirty-three NMC_LRRK2_ individuals met these criteria and fell into this prodromal subgroup (Tables 1, 2, 3, and 4).

**Table 1:** Overview of demographic and clinical data as well as inter-group comparisons for the cohorts IPD, PD_LRRK2_ and CON stratified by gender

	Female *N* = 211			Male *N* = 208			Female				Male			
	IPD *N* = 49	PD_LRRK2_ *N* = 73	CON *N* = 89	IPD *N* = 95	PD_LRRK2_ *N* = 69	CON *N* = 44	*p* value IPD PD_LRRK2_ CON	*p* value Post hoc IPD CON	*p* value Post hoc PD_LRRK2_ CON	*p* value Post hoc IPD PD_LRRK2_	*p* value IPD PD_LRRK2_ CON	*p* value Post hoc IPD CON	*p* value Post hoc PD_LRRK2_ CON	*p* value Post hoc IPD PD_LRRK2_
Age Years	68 46–83	67 35–89	57 18–83	69 46–80	65 36–88	58 28–83	0.001	0.001	0.001	0.999	0.001	0.001	0.012	0.204
Age at onset Years	60 39–77	59 29–76		61 39–75	54 23–82					0.280				0.001
Disease duration Years	7 1–20	10 1–25		7 1–21	11 1–34					0.003				0.001
UPDRS-III	26 6–64	18 2–82	0 0–9	28 9–61	19 1–70	0 0–8	0.001	0.001	0.001	0.379	0.001	0.001	0.001	0.001
HY	2 1–4	2 1–5		2 1–4	2 1–5					0.544				0.889
MoCA	27 11–30	25 6–30	27 17–30	27 13–30	24 5–30	27 17–30	0.001	0.999	0.001	0.014	0.001	0.999	0.002	0.001
RBD Questionnaire	3 0–11	3 0–11	1 0–13	4 0–13	3 0–12	1 0–11	0.001	0.001	0.001	0.941	0.001	0.001	0.017	0.009
ESS	9 1–18	6 0–24	6 0–15	10 0–23	8 0–24	6 0–14	0.002	0.002	0.999	0.024	0.001	0.001	0.246	0.057
SCOPA-AUT	15 2–36	15 0–45	7 0–41	13 0–40	12 0–51	6 0–30	0.001	0.001	0.001	0.999	0.001	0.001	0.001	0.999
Regular anti-inflammatory medication (% of individuals)	12.2	10.3	1.2	10.5	3.1	0.0	0.022	0.010	0.023	0.772	0.032	0.033	0.522	0.125

Data are presented as median with range. *p* values reflect results from Kruskal-Wallis test and, in case of significant differences between the three cohorts, pair-wise post hoc Dunn Test including correction for multiple comparisons. Statistical analysis for the intake of anti-inflammatory medication was done using chi-square test

*IPD* patients with idiopathic Parkinson’s disease, *PD*_*LRRK2*_ patients with Parkinson’s disease carrying a *LRRK2* mutation, *NMC*_*LRRK2*_ non-manifesting *LRRK2* mutation carriers, *CON* healthy control individuals, *ESS* Epworth Sleepiness Scale, *HY* Hoehn and Yahr Scale, *LEDD* levodopa-equivalent daily dosage, *MoCA* Montreal Cognitive Assessment, *RBD* REM sleep behavior disorder, *UPDRS-III* Unified Parkinson’s disease Rating Scale

**Table 2:** Overview of levels of inflammatory-related and neurotrophic markers as well as inter-group comparisons for the cohorts IPD, PD_LRRK2_, and CON stratified by gender

	Female *N* = 211			Male *N* = 208			Female				Male			
Serum marker	IPD *N* = 49	PD_LRRK2_ *N* = 73	CON *N* = 89	IPD *N* = 95	PD_LRRK2_ *N* = 69	CON *N* = 44	*p* value IPD PD_LRRK2_ CON	*p* value Post hoc IPD CON	*p* value Post hoc PD_LRRK2_ CON	*p* value Post hoc IPD PD_LRRK2_	*p* value IPD PD_LRRK2_ CON	*p* value Post hoc IPD CON	*p* value Post hoc PD_LRRK2_ CON	*p* value Post hoc IPD PD_LRRK2_
Alpha fetoprotein ng/ml	0.97 0.09–6.20	0.81 0.12–4.07	0.76 0.07–6.94	1.17 0.14–7.17	0.93 0.12–14.10	0.95 0.33–3.52	0.004	0.008	0.017	0.014	0.034	0.070	0.849	0.032
BDNF (Brain-derived neurotrophic factor) ng/ml	27.40 15.50–40.60	18.90 3.85–55.40	19.70 2.92–41.70	25.50 10.60–56.30	15.60 1.52–37.20	17.05 2.88–45.10	<0.001	<0.001	0.053	<0.001	<0.001	<0.001	0.311	<0.001
ENA-78 (Epithelial-derived neutrophil-activating peptide 78) ng/ml	2.08 0.82–6.15	1.91 0.56–8.36	2.03 0.48–7.70	1.62 0.48–4.61	1.43 0.36–5.08	1.54 0.19–4.77	0.403	0.710	0.499	0.307	0.465	0.439	0.016	0.766
FABP (Fatty-acid-binding protein) ng/ml	4.97 2.24–81.10	3.71 0.40–26.70	2.87 0.97–12.00	5.91 1.45–22.20	4.44 1.37–18.90	4.04 1.44–11.50	<0.001	<0.001	<0.001	<0.001	<0.001	<0.001	0.006	0.002
GH (Growth hormone) ng/ml	1.39 0.11–14.40	2.47 0.08–26.50	1.61 0.11–29.60	0.79 0.04–14.90	0.87 0.08–30.80	0.36 0.07–24.50	0.145	0.326	0.415	0.117	0.004	0.083	0.005	0.030
ICAM-1 (Intercellular adhesion molecule 1) ng/ml	152.62 65.70–224.00	161.00 73.50–262.00	160.00 80.00–288.00	149.00 71.60–273.00	150.00 51.60–246.00	150.50 83.40–232.00	0.340	0.544	0.722	0.018	0.251	0.183	0.529	0.267
IL-1-beta pg/ml	2.18 1.02–4.97	1.83 0.29–4.53	1.84 0.56–4.10	2.57 1.11–17.10	2.38 0.71–5.22	2.17 1.02–3.30	0.052	0.018	0.726	0.239	0.012	0.019	0.179	0.033
IL-4 pg/ml	16.80 8.20–33.20	15.10 5.57–27.00	15.10 6.73–31.50	15.90 5.16–41.20	13.20 4.43–27.10	13.10 5.02–27.00	0.097	0.409	0.083	0.095	0.024	0.070	0.671	0.030
IL-6 pg/ml	4.36 1.67–20.30	4.26 1.27–16.30	3.65 1.27–99.10	5.29 1.67–13.90	5.63 2.00–13.70	4.71 2.17–9.57	0.008	0.017	0.053	0.020	0.033	0.346	0.002	0.254
IL-8 pg/ml	9.72 2.85–38.10	12.90 3.17–60.70	11.60 3.60–60.30	10.70 2.94–771.00	12.30 2.93–209.00	10.75 2.65–77.50	0.052	0.025	0.242	0.232	0.332	0.428	0.145	0.054
IL-10 pg/ml	4.68 1.72–36.10	3.72 1.78–19.20	3.26 1.78–21.80	5.09 2.55–36.90	3.29 1.78–13.80	3.26 1.78–7.59	0.001	<0.001	0.392	0.032	<0.001	<0.001	0.488	<0.001
IL-12-p40 ng/ml	0.29 0.12–0.71	0.21 0.10–0.49	0.24 0.07–0.69	0.25 0.07–0.88	0.22 0.07–0.62	0.16 0.07–0.36	0.007	0.004	0.228	0.016	<0.001	<0.001	0.015	0.059
IL-16 pg/ml	532.00 152.00–1000.00	543.00 237.00–1080.00	551.00 239.00–1370.00	522.00 224.00–1580.00	497.00 232.00–1310.00	520.00 268.00–760.00	0.537	0.265	0.535	0.663	0.611	0.851	0.603	0.403
IL-18 pg/ml	282.00 147.00–546.00	272.00 101.00–752.00	282.00 140.00–3360.00	304.00 137.00–925.00	312.00 133.00–771.00	306.00 141.00–681.00	0.521	0.390	0.725	0.362	0.094	0.812	0.040	0.020
Leptin ng/ml	17.50 4.83–56.20	17.80 0.66–113.00	15.70 2.10–123.00	6.59 0.69–46.00	6.74 0.63–32.40	7.06 1.14–32.50	0.934	0.460	0.624	0.951	0.534	0.781	0.162	0.631
MCP-1 (Monocyte chemotactic protein 1) pg/ml	396.00 177.00–941.00	366.00 152.00–1190.00	342.00 113.00–844.00	448.00 21.90–872.00	333.00 99.90–802.00	323.50 120.00–916.00	0.511	0.268	0.922	0.136	0.066	0.006	0.389	0.115
MDC (Macrophage-derived chemokine) pg/ml	425.00 292.00–676.00	516.00 125.00–964.00	474.00 93.80–1800.00	401.00 171.00–838.00	407.00 146.00–3860.00	483.00 188.00–950.00	0.113	0.289	0.286	0.014	0.014	0.017	0.381	0.036
MIP-1-beta (Macrophage inflammatory protein-1-beta) pg/ml	251.00 70.50–1160.00	217.00 18.40–588.00	215.00 62.20–955.00	237.00 29.70–737.00	207.00 75.90–1530.00	208.00 69.40–2520.00	0.034	0.011	0.097	0.264	0.762	0.536	0.491	0.189
MMP-3 (Matrix-metallo-proteinase-3) ng/ml	14.80 4.42–37.10	13.50 6.31–120.00	12.30 6.08–92.90	23.00 7.28–59.90	24.00 8.01–48.40	23.55 9.61–53.80	0.013	0.052	0.029	0.057	0.594	0.891	0.703	0.283
MMP-9 (Matrix-metallo-proteinase-9) ng/ml	16.80 6.80–28.00	20.90 9.28–46.20	19.60 12.60–61.60	21.20 12.10–42.30	27.00 15.60–121.00	25.40 16.10–37.80	<0.000	<0.001	0.100	<0.001	<0.001	0.222	0.058	<0.001
SCF (Stem cell factor) pg/ml	319.00 169.00–535.00	269.00 78.30–635.00	277.00 117.00–614.00	293.00 154.00–514.00	251.00 81.90–587.00	279.00 127.00–494.00	<0.001	<0.001	0.016	0.009	0.001	0.004	0.344	<0.001
TF (Tissue factor) ng/ml	0.28 0.08–0.72	0.25 0.12–0.67	0.24 0.07–0.53	0.43 0.08–23.80	0.32 0.13–0.74	0.30 0.15–0.43	0.064	0.231	0.002	0.833	0.011	0.057	0.187	0.102
TNF-alpha (Tumor necrosis factor alpha) pg/ml	58.70 11.70–110.00	60.00 21.20–129.00	60.00 27.60–110.00	76.50 39.20–187.00	73.30 30.30–143.00	69.30 49.80–105.00	0.666	0.294	0.925	0.427	0.200	0.299	0.161	0.296
TPO (Thrombopoietin) pg/ml	2.67 1.75–3.55	2.07 0.19–3.76	2.37 0.56–4.48	2.54 1.62–3.68	1.98 0.31–4.08	2.25 0.59–3.64	<0.001	0.002	0.042	0.001	<0.001	<0.001	0.069	<0.001

Data are presented as median with range. *p* values reflect results from inter-group comparisons between IPD, PD_LRRK2_, and CON using multivariate variance analysis including age, regular intake of anti-inflammatory medication, and study site as co-variates. Correction for multiple testing for the number of groups that have been compared (*n* = 3 for IPD, PD_LRRK2_, CON = *p* < 0.016) was done according to Bonferroni and statistical significance was defined as *p* < 0.016. In case of significant differences between the three cohorts, we calculated pair-wise post hoc comparison including age, regular intake of anti-inflammatory medication, and study site as co-variates; and in the case of comparison between IPD and PD_LRRK2_, also disease duration was introduced as co-variate

*IPD* patients with idiopathic Parkinson’s disease, *PD*_*LRRK2*_ patients with Parkinson’s disease carrying a *LRRK2* mutation, *CON* healthy control individuals

**Table 3:** Overview of demographic and clinical data as well as inter-group comparisons for the cohorts NMC_LRRK2_ and CON stratified by gender

	Female *N* = 152		Male *N* = 96		Female	Male
	NMC_LRRK2_ *N* = 63	CON *N* = 89	NMC_LRRK2_ *N* = 52	CON *N* = 44	*p* value NMC_LRRK2_ CON	*p* value NMC_LRRK2_ CON
Age Years	51 23–84	57 18–83	54 27–82	58 28–83	0.087	0.110
UPDRS-III	0 0–13	0 0–9	0 0–15	0 0–8	0.021	0.542
MoCA	28 18–30	27 17–30	27 18–30	27 17–30	0.756	0.809
RBD Questionnaire	1 0–9	1 0–13	2 0–9	1 0–11	0.396	0.943
ESS	6 0–15	6 0–15	5 0–15	6 0–14	0.948	0.399
SCOPA-AUT	8 1–33	7 0–41	7 0–39	6 0–30	0.221	0.729
Regular anti-inflammatory medication (% of individuals)	3.7	1.2	4.6	0.0	0.561	0.495

Data are presented as median with range. *p* values reflect results from Mann–Whitney test. The intake of anti-inflammatory medication is given as % of individuals from the respective cohort; statistical analysis was done using chi-square test

*NMC*_*LRRK2*_ non-manifesting *LRRK2* mutation carriers, *CON* healthy control individuals, *ESS* Epworth Sleepiness Scale, *HY* Hoehn and Yahr Scale, *LEDD* levodopa-equivalent daily dosage, *MoCA* Montreal Cognitive Assessment, *RBD* REM sleep behavior disorder, *UPDRS-III* Unified Parkinson’s disease Rating Scale

**Table 4:** Overview of levels of inflammatory-related and neurotrophic markers as well as inter-group comparisons for the cohorts NMC_LRRK2_ (total and prodromal subgroup) and CON stratified by gender

	Female *N* = 152			Male *N* = 96			Female		Male	
Serum Marker	NMC_LRRK2_ *N* = 63	NMC_LRRK2_ Prodromal *N* = 18	CON *N* = 89	NMC_LRRK2_ *N* = 52	NMC_LRRK2_ Prodromal *N* = 15	CON *N* = 44	*p* value NMC_LRRK2_ CON	*p* value NMC_LRRK2_ Prodromal CON	*p* value NMC_LRRK2_ CON	*p* value NMC_LRRK2_ Prodromal CON
Alpha fetoprotein ng/ml	0.59 0.06–3.53	0.58 0.19–2.28	0.76 0.07–6.94	0.89 0.14–2.47	0.93 0.31–2.47	0.95 0.33–3.52	0.004	0.051	0.068	0.281
BDNF (Brain-derived neurotrophic factor) ng/ml	17.50 3.30–41.70	26.20 3.85–41.70	19.70 2.92–41.70	13.00 1.40–40.50	6.98 1.74–36.60	17.05 2.88–45.10	0.376	0.258	0.026	0.128
ENA-78 (Epithelial-derived neutrophil-activating peptide 78) ng/ml	2.14 0.38–5.77	2.26 0.38–5.76	2.03 0.48–7.70	1.63 0.46–5.98	1.71 0.46–3.75	1.54 0.19–4.77	0.610	0.811	0.016	0.203
FABP (Fatty-acid-binding protein) ng/ml	2.87 0.88–14.40	3.03 1.62–7.99	2.87 0.97–12.00	3.71 2.05–17.80	3.80 2.44–17.80	4.04 1.44–11.50	<0.001	<0.001	<0.001	0.096
GH (Growth hormone) ng/ml	2.18 0.08–35.60	3.16 0.08–30.30	1.61 0.11–29.60	0.35 0.06–6.28	0.36 0.06–1.37	0.34 0.07–24.50	0.109	0.266	0.175	0.270
ICAM-1 (Intercellular adhesion molecule 1) ng/ml	156.00 103.00–317.00	157.50 121.00–224.00	160.00 80.00–288.00	154.50 99.10–329.00	155.00 99.10–329.00	150.50 83.40–232.00	0.839	0.930	0.085	0.632
IL-1-beta pg/ml	1.76 0.71–4.41	2.07 0.94–4.41	1.84 0.56–4.10	2.22 0.78–4.68	2.46 0.78–3.67	2.17 1.02–3.30	0.427	0.072	0.471	0.158
IL-4 pg/ml	15.90 4.89–30.00	15.80 9.01–26.80	15.10 6.73–31.50	13.10 5.02–23.60	14.82 8.19–20.90	14.00 5.02–27.00	0.562	0.458	0.333	0.692
IL-6 pg/ml	3.65 1.05–10.60	3.71 2.11–8.77	3.65 1.27–99.10	4.42 2.50–17.50	4.87 2.50–10.30	4.71 2.17–9.57	0.062	0.039	0.246	0.223
IL-8 pg/ml	10.70 4.89–30.00	10.85 3.17–84.00	11.60 3.60–60.30	10.30 2.36–39.20	10.50 6.53–39.20	10.75 2.65–77.50	<0.001	0.039	0.029	0.288
IL-10 pg/ml	3.57 1.78–32.80	3.61 1.80–9.69	3.26 1.78–7.70	3.44 1.78–9.96	3.49 1.78–9.96	3.26 1.78–7.59	0.910	0.809	0.820	0.932
IL-12-p40 ng/ml	0.24 0.07–0.77	0.24 0.07–0.77	0.24 0.07–0.69	0.19 0.08–0.38	0.22 0.09–0.38	0.16 0.07–0.36	0.010	0.001	0.294	0.432
IL-16 pg/ml	497.00 177.00–752.00	532.50 342.00–735.00	551.00 239.00–1370.00	524.00 261.00–1060.00	549.00 419.00–815.00	520.00 268.00–760.00	0.007	0.197	0.100	0.281
IL-18 pg/ml	288.00 110.00–15000.00	290.00 152.00–440.00	282.00 140.00–3360.00	313.00 165.00–872.00	333.00 186.00–872.00	306.00 141.00–681.00	0.551	0.828	0.083	0.084
Leptin ng/ml	17.10 1.90–90.60	24.25 7.16–44.70	15.70 2.10–123.00	7.03 1.48–38.40	9.90 3.60–31.90	7.06 1.14–32.50	0.674	0.035	0.314	0.475
MCP-1 (Monocyte chemotactic protein 1) pg/ml	333.00 40.80–854.00	325.50 199.00–635.00	342.00 113.00–844.00	329.50 12.20–740.00	292.00 12.20–667.00	323.50 120.00–916.00	0.246	0.631	0.148	0.290
MDC (Macrophage-derived chemokine) pg/ml	452.00 216.00–981.00	539.50 216.00–981.00	474.00 93.80–1800.00	464.00 217.00–855.00	423.00 261.00–774.00	483.00 188.00–950.00	0.514	0.504	0.249	0.692
MIP-1-beta (Macrophage inflammatory protein-1-beta) pg/ml	183.00 61.50–720.00	240.00 61.50–436.00	215.00 62.20–955.00	167.00 77.20–736.00	220.00 77.20–736.00	208.00 69.40–2520.00	0.607	0.302	0.240	0.604
MMP-3 (Matrix-metallo-proteinase-3) ng/ml	10.70 4.04–66.60	12.70 6.32–66.60	12.30 6.08–92.90	21.35 11.30–46.80	21.00 11.90–45.20	23.55 9.61–53.80	<0.001	0.055	0.418	0.600
MMP-9 (Matrix-metallo-proteinase-9) ng/ml	20.40 10.80–40.90	22.45 11.90–40.90	19.60 12.60–61.60	25.70 16.10–36.10	25.30 16.10–36.10	25.40 16.10–37.80	0.009	0.007	0.762	0.931
SCF (Stem cell factor) pg/ml	264.00 154.00–520.00	263.00 193.00–404.00	277.00 117.00–614.00	280.00 145.00–635.00	299.00 190.00–458.00	279.00 127.00–494.00	0.013	0.037	0.076	0.166
TF (Tissue factor) ng/ml	0.24 0.12–0.57	0.26 0.15–0.57	0.24 0.07–0.53	0.29 0.13–1.67	0.32 0.16–0.48	0.30 0.15–0.43	0.004	0.004	0.514	0.464
TNF-alpha (Tumor necrosis factor alpha) pg/ml	54.30 27.60–106.00	55.75 41.60–106.00	60.00 27.60–110.00	74.60 33.00–103.00	78.30 38.40–98.60	69.30 49.80–105.00	0.752	0.652	0.149	0.262
TPO (Thrombopoetin) pg/ml	2.08 0.19–3.86	2.24 0.22–3.49	2.37 0.56–4.48	1.62 0.55–4.18	2.05 0.55–3.87	2.37 0.59–3.64	0.175	0.204	0.082	0.115

Data are presented as median with range. *p* values reflect results from inter-group comparisons between NMC_LRRK2_ and CON using multivariate variance analysis including age, regular intake of anti-inflammatory medication, and study site as co-variates. Correction for multiple testing for the number of groups that have been compared (*n* = 2 for NMC_LRRK2_, CON = *p* < 0.025) was done according to Bonferroni and statistical significance was defined as *p* < 0.025

*NMC*_*LRRK2*_ non-manifesting *LRRK2* mutation carriers, *CON* healthy control individuals

### Clinical investigations

The consortium followed standardized data acquisition protocols to ensure that tests conducted at multiple sites can be pooled. Besides demographics such as gender, age, age at onset, and disease duration, the following clinical parameters were of interest and analyzed in the present study. Diagnosis of Parkinson’s disease was defined according to the UK Brain Bank Criteria with the exception that a positive family history for PD was not considered an exclusion criterion [16]. Severity of motor symptoms was assessed using part III of the MDS-Unified Parkinson’s disease Rating Scale (MDS-UPDRS-III) [17]. Stage of disease was categorized according to the modified Hoehn and Yahr Scale (H&Y) [18]. Cognitive function was tested by the Montreal Cognitive Assessment (MoCA). A cut-off of ≤26 out of 30 points was set to indicate cognitive impairment [19]. A cut-off of ≥5 points in the REM Sleep Behavior Disorder Screening Questionnaire (RBD Questionnaire) was interpreted as presence of RBD [20]. The Epworth Sleepiness Scale (ESS) was used to assess excessive daytime sleepiness [21] and the SCOPA-AUT to assess autonomic dysfunction [22].

### Biomaterial and analyses of immune-associated markers in serum

Serum samples were collected after overnight fasting between 8.00 and 11.00 am, prepared and stored according to standardized operating procedures as defined by the MJFF consortium. Serum was centrifuged at 2000 g, 4 °C for 10 min and stored at −80 °C within 60 min after collection. Levels of 28 immune-associated markers were measured as follows: samples were thawed at room temperature, vortexed, spun at 18.000 × *g* for 1 min and pipetted into a master microtiter plate for multiplexed immunoassay. The kit components of the multiplexed immunoassay were kindly provided by Myriad RBM, Austin, TX, USA (http://rbm.myriad.com). After dilution with assay diluents in a manner of 1:5, an aliquot of 10-μl diluted serum was introduced into one of the capture microsphere multiplexes followed by incubation at room temperature for 1 h. Reporter antibodies were added followed by incubation for an additional hour at room temperature. Streptavidin-phycoerythrin solution was added for development and incubated for 1 h at room temperature. For control purposes, calibrators and controls were included on each microtiter plate. Standard curve, control, and sample QC were performed to ensure proper assay performance (please see Additional file 1: Table S3 for details on LLD, LLOQ, and average concentrations as well as intra- and inter-assay CVs). Samples were tested in singles. Analysis was performed using the Luminex 100/200 instrument and data were interpreted using the software developed and provided by Myriad RBM.

The following 4 serum markers were excluded from analysis due to missing values in >5 % of study participants: IL-1α, IL-7, IL-13, and IL-15. Of the remaining, approximately 1 % of the overall data were missing values, which were replaced by the overall group mean of the respective parameter. A total of 23 immune-associated serum markers as well as BDNF were included in the analyses. For a list of all assessed markers, we refer to Tables 2 and 4.

### Statistics

Statistical analysis was performed using SPSS 22.0 software for Windows SPSS (Inc., Chicago, IL, III, USA). Dichotomous data were analyzed using the chi-square test.

1. Multivariate linear regression analyses stratified by cohort were used to evaluate independent associations of gender, age, disease duration, intake of anti-inflammatory medication, and clinical characteristics with levels of immune markers.
2. To evaluate PD disease status-specific characteristics, we performed inter-group comparisons of demographic, clinical, and immune marker levels between the two PD cohorts and healthy control individuals (IPD, PD_LRRK2_, CON). Due to skewed data, non-parametric testing using Kruskal-Wallis test was performed for the comparison of demographic and clinical data. In case of significant differences in the Kruskal-Wallis test, pair-wise post hoc Dunn test including correction for multiple testing was applied. Levels of immune markers were first normalized by log transformation before inter-group comparisons using multivariate variance analyses corrected for age, intake of anti-inflammatory medication, and study site were done. In case of significant differences in the multivariate variance analysis, pair-wise post hoc comparisons also corrected for age, disease duration (comparison of the two PD cohorts), intake of anti-inflammatory medication, and study site were applied. Correction for multiple testing for the number of groups that have been compared (*n* = 3 for IPD, PD_LRRK2_, CON = *p* < 0.016) was done according to Bonferroni. The pair-wise post hoc comparison between the two PD cohorts (IPD, PD_LRRK2_) further allowed evaluating LRRK2-specific effects in the PD patients.
3. Discriminant analysis in IPD, PD_LRRK2_, and CON including all assessed serum markers was performed to test whether specific immune profiles could discriminate between disease status and genotype and thereby correctly classify individuals to the respective cohort based on the immune marker profile. Discriminant analysis is similar to linear regression by predicting an outcome. The difference between both methods is the classification of the dependent variable which should be categorical when using discriminant analysis (in our case, the three cohorts) as opposed to linear regression where the dependent variable is an interval variable, thereby impeding this method for our analysis. The variable “cohort” was introduced as dependent variable whereas all immune markers were entered at once without prior selection (to ensure an unbiased analysis) as predictors (independent variables). The discriminant analysis weights the effect of all immune markers in order to identify and combine the most important ones which are referred to as discriminant score.
4. To test whether NMC_LRRK2_ present with similar aspects of inflammatory profiles as seen in the PD-affected cohorts, we performed inter-group comparisons of demographic, clinical and immune markers between NMC_LRRK2_ and CON. Due to skewed data, non-parametric testing using Mann–Whitney test was performed for the comparison of demographic and clinical data. Levels of immune markers were first normalized by log transformation before inter-group comparisons using multivariate variance analyses corrected for age were done. Correction for multiple testing for the number of groups that have been compared (*n* = 2 for NMC_LRRK2_ CON = *p* < 0.025) was done according to Bonferroni.

Since regression analysis revealed a significant impact of gender on the immune marker levels and gender was distributed differently across our cohorts, all analyses outlined in this section were done separately stratified by gender.

### Standard protocol approvals, registrations, and patient consents

The study was approved by the respective local ethics committees of the participating centers. All participants gave written informed consent.

## Results

Clinical and demographic characteristics of the cohorts IPD, PD_LRRK2_, and CON are shown in Table 1; the respective data for NMC_LRRK2_ are given in Table 3.

1. Multivariate linear regression analyses stratified by cohort revealed a relevant impact of gender on levels of immune markers. While females had significantly higher levels of ENA-78, GH, IL-4, IL-12-p40, and leptin, males showed higher levels of FABP, IL-1-beta, IL-6, MMP-3, MMP-9, TF, and TNF-alpha. Moreover, discriminant analysis revealed a clear separation between genders based on the immune marker profile in the overall cohort of 534 individuals with a correct classification to the respective gender of 87.8 %. For details, see Additional file 1: Table S2 and Additional file 2: Figure S1.
2. Detailed descriptive data and results of inter-group comparisons including post hoc pair-wise comparison of levels of immune markers between IPD, PD_LRRK2_, and CON are given in Table 2. Compared to CON, females and males of both PD cohorts had significantly higher levels of the pro-inflammatory marker FABP. Moreover, IPD but not PD_LRRK2_ had significantly higher levels of the pro-inflammatory marker IL-12-p40 as well as of the anti-inflammatory marker IL-10, the neurotrophic factor BDNF, and the survival factor SCF compared to CON. Levels of MMP-9 were lowest in IPD compared to PD_LRRK2_.
3. Discriminant analysis in IPD, PD_LRRK2_, and CON including all assessed serum markers revealed FABP (structure matrix coefficient: female = 0.273, male = 0.272), IL-1-beta (structure matrix coefficient: female = 0.220, male = 0.240), IL-12-p40 (structure matrix coefficient: female = 0.230, male = 0.435), MMP-9 (structure matrix coefficient: female = −0.263, male = 0.476), TPO (structure matrix coefficient: female = −0.398, male = 0.412), and BDNF (structure matrix coefficient: female = 0.337, male = 0.544) as the most important factors for discrimination. Overall, 69.7 % of the female and 71.2 % of the male individuals could be correctly classified to the respective cohort based on the immune profile.
4. Detailed descriptive data and results of inter-group comparisons of levels of immune markers between NMC_LRRK2_ and CON are given in Table 4. Female NMC_LRRK2_ who were classified as prodromal subgroup showed increased levels of FABP compared to CON (3.03 vs. 2.87 ng/ml; *p* < 0.001). Overall, NMC_LRRK2_ including the “prodromal subgroup” of NMC_LRRK2_ had similar or even lower levels of immune markers compared to CON and thereby did not present with activated inflammatory profiles as seen in the PD-affected cohorts.

## Discussion

By assessing immune profiles in idiopathic (IPD) and LRRK2-associated PD patients (PD_LRRK2_) as well as in yet non-manifesting LRRK2 mutation carriers (NMC_LRRK2_) and healthy control individuals (CON), we show that (1) levels of inflammatory markers are strongly gender-dependent, (2) both PD-affected cohorts, stratified by gender, present with increased levels of the pro-inflammatory marker FABP, (3) IPD patients, but not PD_LRRK2_, additionally have increased levels of the pro-inflammatory marker IL-12-p40 as well as the anti-inflammatory species IL-10, the neurotrophin BDNF, and the survival factor SCF, and (4) NMC_LRRK2_ present with CON-like profiles, arguing against a relevant role of the inflammatory markers assessed in this study in the prodromal PD_LRRK2_ phase.

It is known that several of the markers assessed in this study show gender-specific differences indicating that immune systems differ substantially between women and men. While females respond to infection and trauma with increased antibody production and anti-inflammatory species such as IL-4 and IL-10, inflammation itself is usually more severe in men resulting in increased mortality in males [23]. In line with the literature and irrespective of the underlying cohort, females in our study presented with higher levels of ENA-78, GH, IL-4, IL-12-p40, and leptin while male participants showed higher levels of IL-1-beta, IL-6, MMP-3, MMP-9, TF, and TNF-alpha [24–28]. Taking these gender differences into consideration is of importance when analyzing immune marker profiles in different cohorts against disease status.

The finding that both PD-affected cohorts presented with significantly increased levels of the pro-inflammatory marker FABP indicates a common disease status-specific pattern irrespective of the underlying genotype. Proteins of the FABP family are abundantly expressed in tissue with fatty acid metabolism such as the brain and the heart. The main function of FABP is the intracellular transport of long-chain fatty acids [29]. It was shown that asyn binds to long-chain fatty acids, resulting in enhanced asyn oligomerization and Lewy body formation in dopaminergic neurons. FABP overexpression aggravated fatty acid-induced asyn oligomerization in a mouse model [30]. Although increased serum and/or CSF levels of FABP have been reported in Lewy body diseases, similar results were also observed in patients with stroke, brain injury, and Creutzfeldt-Jakob disease, suggesting that this marker is rather non-specific [31–33]. Pro-inflammatory IL-12 species including IL-12-p40 are produced by activated macrophages and microglia and enhance T cell proliferation and production of other pro-inflammatory cytokines. IL-12 was previously shown to be increased in serum of PD patients [34].

Interestingly, we also found anti-inflammatory and neurotrophic markers to be higher in idiopathic PD patients than in controls. IL-10 was shown to be neuroprotective in ischemic cell and animal models and was suggested to modulate high levels of the pro-inflammatory cytokine IL-12 in PD patients [34]. BDNF is a widely expressed neurotrophin, which plays a crucial role in neuronal survival. In inflammatory brain lesions, activated monocytes directly secrete bioactive BDNF, which promotes neuronal survival in vitro [13]. Moreover, it was demonstrated that BDNF is positively associated with inflammatory markers in AD [35]. Interestingly, increased serum levels of BDNF have been found in PD patients with longer disease duration and more severe motor impairment [36]. In vivo and in vitro experiments show an up-regulation of the stem cell factor SCF in neurons of injured brain tissue paralleled by neural stem/progenitor cell migration, indicating that SCF is involved in pathways limiting and/or repairing neuronal damage [37].

**Fig. 1:**
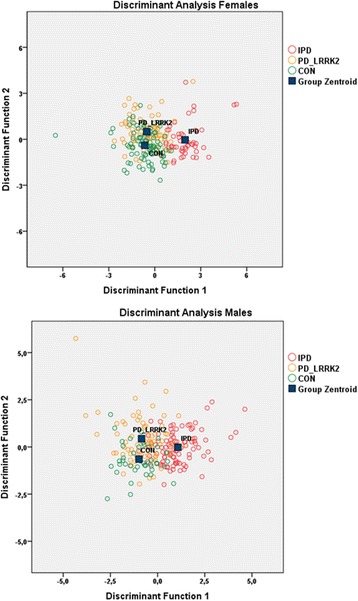
Illustration of the distribution of the discriminant function scores for IPD, PD_LRRK2_, and CON stratified by gender. While IPD patients show little overlap with PD_LRRK2_ and CON indicating a good discrimination based on the immune marker profile, PD_LRRK2_ and CON present a broader overlap suggesting a possibly less robust inflammation in PD_LRRK2_. These findings seem similar across females and males with an overall discrimination between these three cohorts of 69.7 % for females and 72.1 % for males

All these results indicate that inflammatory processes are evident in clinically manifest Parkinson’s disease (potentially to a lesser degree in PD_LRRK2_ as the median levels and numbers of inflammatory markers significantly different from CON were lower in PD_LRRK2_ than in IPD; Table 2, Fig. 1) and are paralleled by increased production of anti-inflammatory and neurotrophic species, possibly as an attempt to counteract tissue damage. However, the exact regulatory mechanisms of pro- and anti-inflammatory pathways need to be further elucidated.

Neither the whole cohort of NMC_LRRK2_ nor the subgroup of “prodromal NMC_LRRK2_” presented with activated inflammatory profiles as seen in the PD-affected cohorts. If we hypothesize that the increased levels of serum immune markers in the PD cohorts reflect aspects of/interaction with the neurodegenerative process, the findings in NMC_LRRK2_ suggest that the activation of the immune system does not start in the very early (prodromal) phase of PD_LRRK2_ in a relevant proportion of individuals but rather after clinical diagnosis. However, it is known that the different prodromal symptoms vary in their predictive value to PD conversion and that speed of progression from prodromal to manifest PD varies among patients and cannot be reliably predicted. Therefore, our cross-sectional results in the prodromal cohort need to be interpreted with caution.

Still, we have to keep in mind that most studies including ours show a large overlap in levels of immune markers comparing cohorts of PD patients and healthy controls. The fact that expression of pro-inflammatory serum markers differs largely even within the cohorts of IPD and PD_LRRK2_ implies that there is a subgroup of individuals in each group, in which concomitant inflammation may play a major role resulting in individual pathophysiologic patterns that could be masked when comparing unselected populations. This needs to be further explored as a possible basis for anti-inflammatory therapeutic strategies. Moreover, the course of neurodegeneration in PD is a dynamic process and most probably not a linear continuum which makes it difficult to estimate when inflammatory processes set in and accelerate. Furthermore, one has to keep in mind that our PD-affected cohorts were older than the control group. Thereby, additional aging-related inflammatory processes known as “inflammaging” might contribute to the inflammatory profiles seen in our PD cohorts.

To overcome limitations of this study, future studies should specifically address the following aspects: (1) relation of serum markers with, e.g., corresponding cerebrospinal fluid markers or (post-mortem) brain material to assess whether these peripheral findings reflect CNS-specific processes, (2) consideration of potential concomitant immune-related diseases and anti-inflammatory therapies in the participants, (3) longitudinal assessment of these inflammatory markers, to evaluate the interrelationship of neurodegeneration and (specific) activation of inflammatory cascades in both IPD and PD_LRRK2_, (4) longitudinal assessment in NMC_LRRK2_ to assess the relationship and time frame between inflammatory markers, prodromal symptoms, and future development of PD. In this context, an accurate definition of “prodromal PD” reflecting a high probability of the individual being in the phase of ongoing neurodegeneration should be applied.

## Conclusions

In conclusion, idiopathic PD patients as well as those with *LRRK2* mutations present with increased levels of the pro-inflammatory serum markers indicating a common disease status-specific pattern. These inflammatory processes are obviously paralleled by anti-inflammatory and neurotrophic processes, possibly as an attempt to counteract tissue damage. Non-manifesting *LRRK2* mutation carriers with symptoms of the prodromal phase of PD did not show activated inflammatory profiles as observed in the PD-affected cohorts, suggesting that the activation of the immune system is not an early event in the disease cascade in at least the majority of this cohort. Finally, levels of inflammatory markers show distinct gender-specific profiles that need to be taken into account when analyzing immune marker profiles in different cohorts against disease status.

## Abbreviations

BDNF, brain-derived neurotrophic factor; CON, healthy control individuals controlled to have no pathogenic *LRRK2* mutation; FABP, fatty-acid-binding protein; IL, interleukin; IPD, idiopathic Parkinson’s disease without *LRRK2* mutation; LRRK2, leucine-rich repeat kinase; NMC_LRRK2_, non-manifesting *LRRK2* mutation carriers; PD, Parkinson’s disease; PD_LRRK2_, Parkinson’s disease with *LRRK2* mutation

## Additional files

Additional file 1: Table S1. Overview of frequency of each *LRRK2* mutation in the respective cohorts. **Table S2.** Overview of results from multivariate linear regression analyses stratified by cohort to illustrate independent associations between gender, age, disease duration, regular intake of anti-inflammatory medication, and clinical characteristics with levels of immune markers. **Table S3.** Overview of quality control of all assessed inflammatory markers (analytes). (DOC 224 kb) Additional file 2: Figure S1. Illustration of the distribution of the discriminant function scores for gender in the whole cohort of 543 individuals. (TIF 2027 kb)

## References

[1] Halliday GM, Stevens CH (2011). Glia: initiators and progressors of pathology in Parkinson’s disease. Mov Disord.

[2] Deleidi M, Gasser T (2013). The role of inflammation in sporadic and familial Parkinson’s disease. Cell Mol Life Sci.

[3] Dzamko N, Geczy CL, Halliday GM (2015). Inflammation is genetically implicated in Parkinson’s disease. Neuroscience.

[4] Schapansky J, Nardozzi JD, LaVoie MJ (2015). The complex relationships between microglia, alpha-synuclein, and LRRK2 in Parkinson’s disease. Neuroscience.

[5] Gagne JJ, Power MC (2010). Anti-inflammatory drugs and risk of Parkinson disease: a meta-analysis. Neurology.

[6] Whitton PS (2010). Neuroinflammation and the prospects for anti-inflammatory treatment of Parkinson’s disease. Curr Opin Investig Drugs.

[7] Glass CK, Saijo K, Winner B, Marchetto MC, Gage FH (2010). Mechanisms underlying inflammation in neurodegeneration. Cell.

[8] Holmans P, Moskvina V, Jones L, Sharma M, Vedernikov A, Buchel F, Saad M, Bras JM, Bettella F, International Parkinson’s Disease Genomics C (2013). A pathway-based analysis provides additional support for an immune-related genetic susceptibility to Parkinson’s disease. Hum Mol Genet.

[9] Lee HJ, Suk JE, Patrick C, Bae EJ, Cho JH, Rho S, Hwang D, Masliah E, Lee SJ (2010). Direct transfer of alpha-synuclein from neuron to astroglia causes inflammatory responses in synucleinopathies. J Biol Chem.

[10] Healy DG, Falchi M, O’Sullivan SS, Bonifati V, Durr A, Bressman S, Brice A, Aasly J, Zabetian CP, Goldwurm S (2008). Phenotype, genotype, and worldwide genetic penetrance of LRRK2-associated Parkinson’s disease: a case–control study. Lancet Neurol.

[11] Brockmann K, Groger A, Di Santo A, Liepelt I, Schulte C, Klose U, Maetzler W, Hauser AK, Hilker R, Gomez-Mancilla B (2011). Clinical and brain imaging characteristics in leucine-rich repeat kinase 2-associated PD and asymptomatic mutation carriers. Mov Disord.

[12] Hakimi M, Selvanantham T, Swinton E, Padmore RF, Tong Y, Kabbach G, Venderova K, Girardin SE, Bulman DE, Scherzer CR (2011). Parkinson’s disease-linked LRRK2 is expressed in circulating and tissue immune cells and upregulated following recognition of microbial structures. J Neural Transm.

[13] Kerschensteiner M, Gallmeier E, Behrens L, Leal VV, Misgeld T, Klinkert WE, Kolbeck R, Hoppe E, Oropeza-Wekerle RL, Bartke I (1999). Activated human T cells, B cells, and monocytes produce brain-derived neurotrophic factor in vitro and in inflammatory brain lesions: a neuroprotective role of inflammation?. J Exp Med.

[14] Berg D, Postuma RB, Adler CH, Bloem BR, Chan P, Dubois B, Gasser T, Goetz CG, Halliday G, Joseph L (2015). MDS research criteria for prodromal Parkinson’s disease. Mov Disord.

[15] Doty RL, Bromley SM, Stern MB (1995). Olfactory testing as an aid in the diagnosis of Parkinson’s disease: development of optimal discrimination criteria. Neurodegeneration.

[16] Litvan I, Bhatia KP, Burn DJ, Goetz CG, Lang AE, McKeith I, Quinn N, Sethi KD, Shults C, Wenning GK (2003). Movement Disorders Society Scientific Issues Committee report: SIC Task Force appraisal of clinical diagnostic criteria for Parkinsonian disorders. Mov Disord.

[17] Goetz CG, Tilley BC, Shaftman SR, Stebbins GT, Fahn S, Martinez-Martin P, Poewe W, Sampaio C, Stern MB, Dodel R (2008). Movement Disorder Society-sponsored revision of the Unified Parkinson’s Disease Rating Scale (MDS-UPDRS): scale presentation and clinimetric testing results. Mov Disord.

[18] Goetz CG, Poewe W, Rascol O, Sampaio C, Stebbins GT, Counsell C, Giladi N, Holloway RG, Moore CG, Wenning GK (2004). Movement Disorder Society Task Force report on the Hoehn and Yahr staging scale: status and recommendations. Mov Disord.

[19] Hoops S, Nazem S, Siderowf AD, Duda JE, Xie SX, Stern MB, Weintraub D (2009). Validity of the MoCA and MMSE in the detection of MCI and dementia in Parkinson disease. Neurology.

[20] Stiasny-Kolster K, Mayer G, Schafer S, Moller JC, Heinzel-Gutenbrunner M, Oertel WH (2007). The REM sleep behavior disorder screening questionnaire—a new diagnostic instrument. Mov Disord.

[21] Johns MW (1991). A new method for measuring daytime sleepiness: the Epworth sleepiness scale. Sleep.

[22] Visser M, Marinus J, Stiggelbout AM, Van Hilten JJ (2004). Assessment of autonomic dysfunction in Parkinson’s disease: the SCOPA-AUT. Mov Disord.

[23] Fairweather D, Frisancho-Kiss S, Rose NR (2008). Sex differences in autoimmune disease from a pathological perspective. Am J Pathol.

[24] Xiong X, Xu L, Wei L, White RE, Ouyang YB, Giffard RG (2015). IL-4 is required for sex differences in vulnerability to focal ischemia in mice. Stroke.

[25] Aomatsu M, Kato T, Kasahara E, Kitagawa S (2013). Gender difference in tumor necrosis factor-alpha production in human neutrophils stimulated by lipopolysaccharide and interferon-gamma. Biochem Biophys Res Commun.

[26] Ramsey JM, Schwarz E, Guest PC, van Beveren NJ, Leweke FM, Rothermundt M, Bogerts B, Steiner J, Ruta L, Baron-Cohen S, Bahn S (2012). Molecular sex differences in human serum. PLoS One.

[27] Manicourt DH, Fujimoto N, Obata K, Thonar EJ (1994). Serum levels of collagenase, stromelysin-1, and TIMP-1. Age- and sex-related differences in normal subjects and relationship to the extent of joint involvement and serum levels of antigenic keratan sulfate in patients with osteoarthritis. Arthritis Rheum.

[28] Woodrum DT, Ford JW, Ailawadi G, Pearce CG, Sinha I, Eagleton MJ, Henke PK, Stanley JC, Upchurch GR (2005). Gender differences in rat aortic smooth muscle cell matrix metalloproteinase-9. J Am Coll Surg.

[29] Coe NR, Bernlohr DA (1998). Physiological properties and functions of intracellular fatty acid-binding proteins. Biochim Biophys Acta.

[30] Shioda N, Yabuki Y, Kobayashi Y, Onozato M, Owada Y, Fukunaga K (2014). FABP3 protein promotes alpha-synuclein oligomerization associated with 1-methyl-1,2,3,6-tetrahydropiridine-induced neurotoxicity. J Biol Chem.

[31] Zimmermann-Ivol CG, Burkhard PR, Le Floch-Rohr J, Allard L, Hochstrasser DF, Sanchez JC (2004). Fatty acid binding protein as a serum marker for the early diagnosis of stroke: a pilot study. Mol Cell Proteomics.

[32] Guillaume E, Zimmermann C, Burkhard PR, Hochstrasser DF, Sanchez JC (2003). A potential cerebrospinal fluid and plasmatic marker for the diagnosis of Creutzfeldt-Jakob disease. Proteomics.

[33] Wada-Isoe K, Imamura K, Kitamaya M, Kowa H, Nakashima K (2008). Serum heart-fatty acid binding protein levels in patients with Lewy body disease. J Neurol Sci.

[34] Rentzos M, Nikolaou C, Andreadou E, Paraskevas GP, Rombos A, Zoga M, Tsoutsou A, Boufidou F, Kapaki E, Vassilopoulos D (2009). Circulating interleukin-10 and interleukin-12 in Parkinson’s disease. Acta Neurol Scand.

[35] Faria MC, Goncalves GS, Rocha NP, Moraes EN, Bicalho MA, Gualberto Cintra MT, Jardim de Paula J, de Miranda LF JR, Clayton de Souza Ferreira A, Teixeira AL (2014). Increased plasma levels of BDNF and inflammatory markers in Alzheimer’s disease. J Psychiatr Res.

[36] Scalzo P, Kummer A, Bretas TL, Cardoso F, Teixeira AL (2010). Serum levels of brain-derived neurotrophic factor correlate with motor impairment in Parkinson’s disease. J Neurol.

[37] Sun L, Lee J, Fine HA (2004). Neuronally expressed stem cell factor induces neural stem cell migration to areas of brain injury. J Clin Invest.

