# Supplementary material for: Inflammatory profile in LRRK2-associated prodromal and clinical PD
Source: J Neuroinflammation. 2016 May 24;13:122. doi: 10.1186/s12974-016-0588-5 (PMC4879729; doi:10.1186/s12974-016-0588-5)
Supplement: Additional file 1: Table S1. — Overview of frequency of each LRRK2 mutation in the respective cohorts. Table S2. Overview of results from multivariate linear regression analyses stratified by cohort to illustrate independent associations between gender, age, disease duration, regular intake of anti-inflammatory medication, and clinical characteristics with levels of immune markers. Table S3. Overview of quality control of all assessed inflammatory markers (analytes). (DOC 224 kb) [file 12974_2016_588_MOESM1_ESM.doc]

**Supplemental Table 1: Overview of frequency of each *LRRK2*** mutation in the respective cohorts.

| **Mutation** | **PD_LRRK2_** | **NMC_LRRK2_** | **NMC_LRRK2-prodromal_** | **p-Value**  **PD_LRRK2_**  **NMC_LRRK2_** | **p-Value**  **PD_LRRK2_**  **NMC_LRRK2-prodromal_** |
| --- | --- | --- | --- | --- | --- |
| p.G2019S  (%) | 79.6 | 68.7 | 72.8 | 0.060 | 0.482 |
| p.R1441G  (%) | 16.2 | 27.0 | 24.2 | 0.050 | 0.312 |
| p.R1441C  (%) | 0.7 | 2.6 | 0 | 0.328 | 0.999 |
| p.N1437H  (%) | 1.4 | 1.7 | 3.0 | 0.999 | 0.468 |
| p.I2020T  (%) | 2.1 | 0 | 0 | 0.255 | 0.999 |

The different mutations seem rather equally distributed among the *LRRK2* cohorts with p.G2019S and p.R1441G together accounting for over 95% of all mutations in each cohort.

**Supplemental Table 2: Overview of results from multivariate linear regression analyses stratified by cohort to illustrate independent associations between gender, age, disease duration, regular intake of anti-inflammatory medication, and clinical characteristics with** levels of immune markers.

|  |  | **Alpha Fetoprotein** | **BDNF** | **ENA-78** | **FABP** | **GH** | **ICAM-1** | **IL-1-beta** | **IL-4** | **IL-6** | **IL-8** | **IL-10** | **IL-12-p40** | **IL-16** | **IL-18** | **Leptin** | **MCP-1** | **MDC** | **MIP-1-beta** | **MMP-3** | **MMP-9** | **SCF** | **TF** | **TNF-alpha** | **TPO** |
| --- | --- | --- | --- | --- | --- | --- | --- | --- | --- | --- | --- | --- | --- | --- | --- | --- | --- | --- | --- | --- | --- | --- | --- | --- | --- |
| **IPD** | **Gender** | 0.047 | -0.124 | **-0.289**  ******* | -0.075 | -0.135 | -0.077 | **0.188*** | -0.004 | 0.119 | 0.079 | 0.019 | -0.042 | 0.046 | 0.131 | **-0.521**  ******* | 0.050 | **-0.189**  ***** | -0.145 | **0.491**  ******* | **0.435**  ******* | -0.162 | 0.110 | **0.338**  ******* | -0.122 |
|  | **Age** | 0.158 | -0.071 | 0.003 | 0.168 | -0.104 | **0.203**  ***** | 0.002 | -0.060 | 0.155 | -0.040 | -0.063 | 0.080 | 0.093 | -0.009 | 0.028 | -0.041 | -0.090 | 0.106 | 0.116 | 0.137 | **0.184**  ***** | 0.009 | 0.136 | 0.006 |
|  | **Disease Duration** | 0.009 | -0.173 | 0.137 | 0.037 | 0.034 | -0.087 | -0.059 | 0.013 | 0.092 | **0.185**  ***** | 0.003 | 0.084 | 0.044 | 0.073 | 0.021 | 0.157 | 0.163 | 0.039 | -0.058 | -0.050 | 0.099 | 0.034 | 0.022 | -0.130 |
|  | **H&Y** | 0.001 | -0.014 | 0.068 | -0.060 | 0.013 | -0.033 | -0.079 | 0.102 | 0.067 | -0.031 | 0.060 | -0.169 | -0.071 | 0.150 | 0.002 | -0.025 | -0.080 | -0.051 | 0.122 | -0.092 | -0.045 | 0.168 | -0.101 | -0.029 |
|  | **UPDRS-III** | -0.105 | 0.043 | -0.129 | -0.022 | 0.052 | **0.216**  ***** | 0.078 | -0.161 | -0.136 | -0.084 | -0.067 | -0.066 | -0.071 | 0.022 | 0.123 | -0.110 | 0.055 | 0.130 | -0.104 | 0.051 | -0.097 | **-0.246**  ***** | 0.072 | -0.102 |
|  | **MoCA** | 0.062 | 0.056 | -0.039 | -0.065 | 0.113 | 0.043 | 0.057 | -0.066 | -0.031 | -0.015 | -0.077 | 0.018 | 0.055 | 0.161 | 0.044 | -0.021 | -0.160 | -0.005 | 0.083 | 0.041 | -0.050 | -0.059 | 0.001 | -0.079 |
|  | **Anti-infl. Medication** | -0.031 | -0.033 | 0.068 | 0.068 | 0.084 | -0.093 | -0.009 | -0.005 | 0.002 | -0.038 | 0.020 | -0.061 | 0.010 | -0.018 | 0.119 | -0.045 | -0.033 | -0.068 | 0.056 | 0.049 | -0.090 | -0.018 | 0.050 | -0.040 |
| **PD_LRRK2_** | **Gender** | 0.127 | -0.143 | **-0.216**  ***** | **0.176**  ***** | -0.087 | -0.167 | **0.270**  ****** | -0.147 | **0.258**  ****** | 0.039 | -0.060 | -0.032 | -0.127 | 0.125 | **-0.463**  ******* | -0.084 | -0.059 | -0.012 | **0.346**  ******* | **0.376**  ******* | -0.043 | **0.285**  ******* | **0.350**  ******* | -0.099 |
|  | **Age** | 0.089 | -0.072 | -0.082 | **0.380**  ******* | 0.180 | 0.097 | -0.067 | 0.058 | 0.168 | 0.012 | -0.085 | 0.040 | 0.082 | 0.065 | 0.086 | -0.175 | 0.003 | -0.176 | 0.150 | 0.099 | 0.077 | 0.169 | 0.039 | -0.114 |
|  | **Disease Duration** | 0.124 | -0.024 | 0.006 | -0.042 | 0.076 | 0.124 | -0.009 | -0.16 | 0.018 | -0.110 | -0.080 | 0.118 | 0.112 | 0.109 | -0.151 | 0.036 | -0.120 | 0.040 | -0.147 | 0.077 | -0.048 | 0.005 | -0.038 | 0.028 |
|  | **H&Y** | -0.033 | -0.157 | -0.148 | 0.009 | -0.131 | -0.002 | 0.120 | 0.036 | 0.017 | 0.117 | -0.005 | 0.091 | 0.112 | -0.040 | 0.031 | -0.111 | 0.079 | 0.174 | 0.187 | 0.205 | -0.034 | 0.219 | 0.144 | -0.212 |
|  | **UPDRS-III** | -0.057 | 0.065 | 0.005 | 0.066 | -0.157 | 0.110 | -0.061 | -0.80 | 0.234 | -0.002 | **0.281**  ***** | -0.074 | -0.116 | -0.118 | -0.012 | -0.034 | 0.041 | 0.003 | 0.014 | 0.067 | 0.060 | -0.117 | -0.027 | 0.082 |
|  | **MoCA** | 0.072 | 0.016 | -0.081 | 0.116 | 0.049 | 0.167 | -0.124 | -0.125 | 0.073 | -0.013 | 0.048 | 0.189 | **0.223**  ***** | 0.061 | 0.157 | 0.018 | 0.129 | 0.079 | -0.020 | 0.124 | -0.035 | -0.052 | 0.033 | -0.030 |
|  | **Anti-infl. Medication** | 0.115 | **0.230**  ***** | 0.109 | 0.138 | 0.014 | -0.068 | 0.074 | -0.010 | 0.009 | -0.046 | 0.037 | -0.005 | -0.003 | -0.026 | **-0.183**  ***** | -0.039 | 0.009 | 0.043 | 0.064 | 0.067 | 0.150 | 0.046 | -0.016 | 0.119 |
| **NMC_LRRK2_** | **Gender** | 0.118 | **-0.207**  ***** | **-0.250**  ****** | **0.201**  ***** | **-0.401**  ******* | -0.093 | 0.128 | **-0.276**  ****** | **0.305**  ****** | -0.001 | -0.020 | **-0.256**  ****** | 0.130 | -0.066 | **-0.547**  ******* | -0.060 | -0.088 | -0.062 | **0.678**  ******* | **0.450**  ******* | 0.005 | **0.245**  ***** | **0.428**  ******* | -0.040 |
|  | **Age** | 0.137 | -0.023 | 0.154 | **0.552**  ******* | -0.105 | 0.149 | 0.064 | -0.041 | 0.138 | **0.431**  ******* | 0.077 | -0.107 | 0.145 | 0.155 | 0.076 | -0.064 | 0.120 | 0.016 | 0.108 | 0.102 | **0.312**  ****** | 0.053 | 0.040 | -0.003 |
|  | **Anti-infl. Medication** | 0.036 | -0.087 | -0.155 | **0.221**  ***** | -0.070 | 0.143 | -0.018 | 0.100 | **0.214**  ***** | -0.169 | -0.009 | 0.004 | **0.221**  ***** | **0.222**  ***** | 0.058 | 0.005 | 0.048 | -0.036 | 0.064 | 0.151 | -0.045 | 0.024 | 0.114 | -0.097 |
| **CON** | **Gender** | -0.003 | -0.067 | -0.162 | **0.271**  ******* | **-0.225**  ***** | -0.174 | **0.238**  ****** | **-0.209**  ***** | **-0.215**  ***** | -0.084 | -0.065 | **-0.330**  ******* | -0.078 | 0.086 | **-0.463**  ******* | -0.057 | -0.037 | 0.105 | **0.492**  ******* | **0.349**  ******* | -0.004 | **0.204**  ***** | **0.348**  ******* | -0.016 |
|  | **Age** | 0.168 | -0.077 | -0.097 | **0.376**  ******* | **-0.222**  ***** | 0.027 | 0.048 | 0.098 | **0.208**  ***** | **0.189**  ***** | -0.016 | **-0.219**  ****** | 0.036 | 0.074 | 0.142 | -0.052 | **-0.181**  ***** | 0.124 | **0.163**  ***** | 0.121 | 0.081 | **0.293**  ******* | 0.135 | -0.153 |
|  | **Anti-infl. Medication** | -0.094 | 0.070 | 0.077 | -0.007 | -0.073 | -0.075 | -0.172 | 0.106 | **-0.205**  ***** | 0.069 | 0.093 | **0.385***** | 0.124 | 0.039 | -0.144 | 0.030 | 0.046 | 0.096 | -0.087 | -0.122 | 0.103 | **-0.170**  ***** | -0.130 | 0.090 |

Data are given as standardized regression coefficient beta.

Statistically significant p-values are in bold and indicated as: * = p ≤ 0.05; ** = p ≤ 0.01; *** = p ≤ 0.001.

**Supplemental Table 3: Overview of quality control of all assessed inflammatory markers (analytes).**

| **Analyte** | **Units** | **LLD** | **LLOQ** |  | **Average Concentration** | **Run 1** | **Run 2** | **Run 3** | **Run 4** | **Run 5** | **Inter-Run** |
| --- | --- | --- | --- | --- | --- | --- | --- | --- | --- | --- | --- |
|  |  |  |  |  |  | **Intra-Assay-CV** | | | | | **Inter-Assay-CV** |
| **Alpha Fetoprotein** | **ng/ml** | 0.034 | 0.276 | Level 1 | 2.45 | 3% | 2% | 3% | 4% | 6% | 13% |
|  |  |  |  | Level 2 | 6.88 | 6% | 2% | 4% | 2% | 4% | 7% |
|  |  |  |  | Level 3 | 34.0 | 2% | 3% | 2% | 6% | 3% | 5% |
| **Brain-Der Neu Fac** | **ng/ml** | 0.017 | 0.058 | Level 1 | 0.53 | 10% | 1% | 5% | 7% | 1% | 6% |
|  |  |  |  | Level 2 | 1.98 | 3% | 12% | 11% | 8% | 0% | 8% |
|  |  |  |  | Level 3 | 15.8 | 8% | 1% | 0% | 1% | 2% | 4% |
| **ENA 78** | **ng/ml** | 0.014 | 0.048 | Level 1 | 0.33 | 4% | 4% | 2% | 3% | 0% | 4% |
|  |  |  |  | Level 2 | 1.32 | 1% | 2% | 2% | 0% | 1% | 4% |
|  |  |  |  | Level 3 | 6.97 | 0% | 0% | 3% | 1% | 1% | 3% |
| **FABP** | **ng/ml** | 0.237 | 1.550 | Level 1 | 17.80 | 7% | 6% | 2% | 1% | 1% | 8% |
|  |  |  |  | Level 2 | 51.60 | 3% | 4% | 0% | 2% | 5% | 4% |
|  |  |  |  | Level 3 | 195.00 | 3% | 0% | 1% | 0% | 1% | 2% |
| **GH** | **ng/ml** | 0.026 | 0.091 | Level 1 | 0.857 | 3% | 15% | 1% | 7% | 11% | 13% |
|  |  |  |  | Level 2 | 1.96 | 4% | 1% | 4% | 8% | 10% | 6% |
|  |  |  |  | Level 3 | 10.00 | 3% | 0% | 2% | 2% | 1% | 2% |
| **ICAM-1** | **ng/ml** | 0.153 | 1.610 | Level 1 | 16.30 | 8% | 2% | 1% | 2% | 0% | 9% |
|  |  |  |  | Level 2 | 43.90 | 1% | 1% | 2% | 0% | 5% | 3% |
|  |  |  |  | Level 3 | 124.00 | 1% | 1% | 2% | 1% | 4% | 5% |
| **IL-1beta** | **pg/ml** | 0.270 | 0.895 | Level 1 | 12.00 | 2% | 8% | 3% | 0% | 3% | 7% |
|  |  |  |  | Level 2 | 39.70 | 4% | 3% | 0% | 4% | 6% | 5% |
|  |  |  |  | Level 3 | 137.00 | 1% | 3% | 3% | 3% | 7% | 4% |
| **IL-4** | **pg/mL** | 4.040 | 13.100 | Level 1 | 117.00 | 6% | 3% | 12% | 5% | 0% | 7% |
|  |  |  |  | Level 2 | 543.00 | 1% | 3% | 7% | 5% | 1% | 5% |
|  |  |  |  | Level 3 | 2587.00 | 7% | 3% | 0% | 1% | 4% | 5% |
| **IL-6** | **pg/ml** | 0.620 | 2.160 | Level 1 | 17.20 | 9% | 13% | 3% | 3% | 3% | 6% |
|  |  |  |  | Level 2 | 73.70 | 7% | 0% | 2% | 1% | 8% | 6% |
|  |  |  |  | Level 3 | 274.00 | 0% | 0% | 5% | 4% | 7% | 7% |
| **IL-8** | **pg/mL** | 2.050 | 6.850 | Level 1 | 65.40 | 0% | 6% | 4% | 5% | 7% | 9% |
|  |  |  |  | Level 2 | 333.00 | 2% | 1% | 8% | 0% | 3% | 6% |
|  |  |  |  | Level 3 | 1659.00 | 1% | 1% | 5% | 3% | 2% | 6% |
| **IL-10** | **pg/mL** | 1.680 | 5.700 | Level 1 | 26.40 | 12% | 2% | 6% | 16% | 3% | 11% |
|  |  |  |  | Level 2 | 220.00 | 2% | 1% | 11% | 2% | 3% | 6% |
|  |  |  |  | Level 3 | 1071.00 | 3% | 2% | 1% | 8% | 6% | 6% |
| **IL-12 p40** | **ng/ml** | 0.055 | 0.185 | Level 1 | 1.42 | 1% | 8% | 5% | 5% | 7% | 5% |
|  |  |  |  | Level 2 | 7.69 | 1% | 0% | 0% | 3% | 1% | 4% |
|  |  |  |  | Level 3 | 40.00 | 1% | 0% | 6% | 7% | 2% | 6% |
| **IL-16** | **pg/mL** | 6.450 | 30.700 | Level 1 | 202.00 | 3% | 1% | 2% | 3% | 2% | 6% |
|  |  |  |  | Level 2 | 818.00 | 1% | 0% | 7% | 1% | 1% | 4% |
|  |  |  |  | Level 3 | 4277.00 | 3% | 2% | 3% | 3% | 4% | 4% |
| **IL-18** | **pg/mL** | 8.750 | 31.000 | Level 1 | 148.00 | 5% | 3% | 9% | 10% | 6% | 11% |
|  |  |  |  | Level 2 | 1108.00 | 8% | 2% | 2% | 5% | 6% | 6% |
|  |  |  |  | Level 3 | 4974.00 | 1% | 1% | 0% | 5% | 4% | 6% |
| **Leptin** | **ng/ml** | 0.025 | 0.151 | Level 1 | 0.80 | 2% | 4% | 2% | 6% | 2% | 8% |
|  |  |  |  | Level 2 | 3.71 | 2% | 1% | 6% | 1% | 2% | 7% |
|  |  |  |  | Level 3 | 13.10 | 2% | 2% | 1% | 4% | 3% | 7% |
| **MCP-1** | **pg/mL** | 5.250 | 21.700 | Level 1 | 95.10 | 2% | 17% | 4% | 5% | 3% | 7% |
|  |  |  |  | Level 2 | 472.00 | 1% | 1% | 4% | 4% | 6% | 4% |
|  |  |  |  | Level 3 | 2364.00 | 2% | 1% | 3% | 5% | 4% | 4% |
| **MDC** | **pg/mL** | 4.390 | 14.600 | Level 1 | 67.50 | 5% | 1% | 2% | 5% | 0% | 6% |
|  |  |  |  | Level 2 | 467.00 | 6% | 1% | 4% | 2% | 6% | 4% |
|  |  |  |  | Level 3 | 1864.00 | 0% | 9% | 3% | 2% | 0% | 8% |
| **MIP-1 beta** | **pg/mL** | 9.250 | 34.100 | Level 1 | 367.00 | 3% | 3% | 1% | 9% | 3% | 6% |
|  |  |  |  | Level 2 | 1271.00 | 3% | 5% | 12% | 2% | 10% | 8% |
|  |  |  |  | Level 3 | 7153.00 | 2% | 2% | 3% | 1% | 7% | 7% |
| **MMP-3** | **ng/ml** | 0.032 | 0.106 | Level 1 | 0.78 | 2% | 3% | 5% | 1% | 0% | 11% |
|  |  |  |  | Level 2 | 5.51 | 3% | 2% | 3% | 1% | 3% | 7% |
|  |  |  |  | Level 3 | 19.30 | 1% | 6% | 4% | 5% | 9% | 6% |
| **MMP-9** | **ng/ml** | 1.720 | 19.600 | Level 1 | 36.60 | 13% | 10% | 9% | 13% | 0% | 14% |
|  |  |  |  | Level 2 | 100.00 | 7% | 1% | 4% | 3% | 7% | 8% |
|  |  |  |  | Level 3 | 871.00 | 1% | 4% | 0% | 0% | 1% | 3% |
| **SCF** | **pg/mL** | 19.900 | 65.500 | Level 1 | 449.00 | 10% | 0% | 5% | 0% | 10% | 7% |
|  |  |  |  | Level 2 | 4143.00 | 0% | 5% | 6% | 1% | 9% | 4% |
|  |  |  |  | Level 3 | 9968.00 | 2% | 3% | 0% | 5% | 2% | 4% |
| **TF** | **ng/ml** | 0.067 | 0.224 | Level 1 | 0.86 | 1% | 3% | 10% | 4% | 0% | 9% |
|  |  |  |  | Level 2 | 3.83 | 4% | 0% | 1% | 2% | 6% | 7% |
|  |  |  |  | Level 3 | 28.40 | 0% | 5% | 0% | 6% | 2% | 5% |
| **TNF-alpha** | **pg/ml** | 2.270 | 8.950 | Level 1 | 141.00 | 5% | 4% | 9% | 5% | 4% | 9% |
|  |  |  |  | Level 2 | 468.00 | 3% | 5% | 1% | 1% | 5% | 6% |
|  |  |  |  | Level 3 | 1760.00 | 4% | 0% | 1% | 7% | 7% | 8% |
| **TPO** | **ng/ml** | 0.048 | 1.050 | Level 1 | 5.64 | 5% | 5% | 1% | 4% | 4% | 4% |
|  |  |  |  | Level 2 | 16.50 | 1% | 3% | 1% | 4% | 4% | 5% |
|  |  |  |  | Level 3 | 77.30 | 4% | 3% | 2% | 0% | 2% | 7% |

LLD = lowest limit of detection; LLOQ = lowest limit of quantification; CV = Coefficients of Variability.
