# Supplementary figures and images for: Inflammatory profile in LRRK2-associated prodromal and clinical PD
Source: J Neuroinflammation. 2016 May 24;13:122. doi: 10.1186/s12974-016-0588-5 (PMC4879729; doi:10.1186/s12974-016-0588-5)

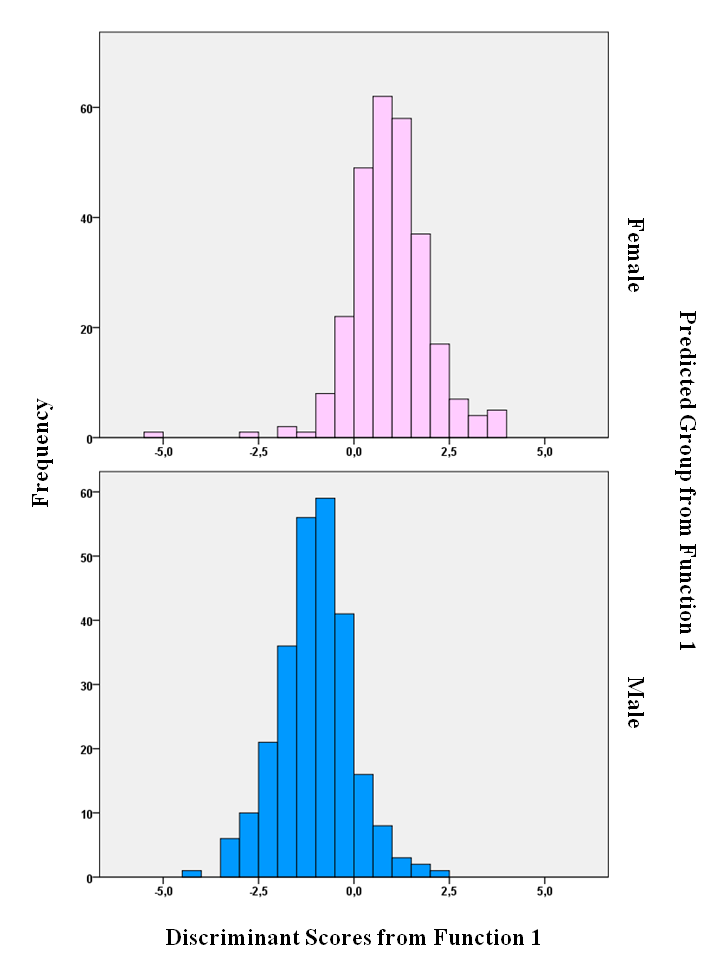

Supplement: Additional file 2: Figure S1. — Illustration of the distribution of the discriminant function scores for gender in the whole cohort of 543 individuals. (TIF 2027 kb) [file 12974_2016_588_MOESM2_ESM.tif]
